# Paternal age, body mass index, and semen volume are associated with chromosomal aberrations-related miscarriages in couples that underwent treatment by assisted reproductive technology

**DOI:** 10.18632/aging.103151

**Published:** 2020-05-08

**Authors:** Zhiyuan Wang, Xiaocong Liu, Jiawei Xu, Qingling Yang, Wenbin Niu, Shanjun Dai, Linli Hu, Yihong Guo

**Affiliations:** 1 Center for Reproductive Medicine, The First Affiliated Hospital of Zhengzhou University, Zhengzhou, China; 2 Henan Province Key Laboratory of Reproduction and Genetics, Henan, China; 3 Department of Preimplantation Genetic Diagnosis, Center for Reproductive Medicine, The First Affiliated Hospital of Zhengzhou University, Zhengzhou, China

**Keywords:** paternal age, body mass index (BMI), semen parameters, miscarried fetus, chromosome karyotype

## Abstract

We investigated the effects of paternal characteristics, including age, body mass index (BMI), and semen parameters on chromosomal aberration-related miscarriages in couples that underwent treatment with assisted reproductive technology (ART). Single nucleotide polymorphism (SNP) array analysis showed chromosomal aberrations in 60.2% (557/925) of miscarried fetuses, including trisomy in 73.1% (407/557) of cases. There were higher chromosomal aberration rates in fetuses for men aged 20-24 years and ≥30 years compared with controls. After adjusting for age and BMI of the female partners, and the BMI and semen parameters of the males, there was no statistically significant effect of paternal age ≥30 years on the risk of chromosomal aberrations-related miscarriages. However, the odds of chromosomal abnormality-related miscarriage were 148% higher for the youngest fathers (age: 20-24 years) than fathers aged 25-29 years [adjusted odds ratio (OR): 2.48, 95% confidence interval (CI): 1.03-5.96; P=0.042]. Furthermore, high male BMI (adjusted OR: 1.56, 95% CI: 1.14-2.14; P=0.005) and low semen volume (adjusted OR: 2.09, 95% CI: 1.06-4.11; P=0.034) were associated with increased risk of chromosomal aberration-related miscarriages. These findings demonstrate that very young paternal age, high BMI, and low semen volume are associated with increased risk of chromosomal aberration-related miscarriages in couples undergoing ART treatment.

## INTRODUCTION

Infertility affects 10-15% of couples globally [1], with paternal factors accounting for nearly 50% of infertility cases [2]. Assisted reproductive technologies (ARTs) help couples to produce a viable embryo that can potentially grow into a healthy offspring by fertilizing the oocyte with spermatozoa of the male partner with infertility issues. However, spontaneous pregnancy loss after ART can result in physical and psychological trauma, and significant economic losses. Previous studies show that 50% of the pregnancy losses are because of embryonic chromosomal abnormalities [3]. Therefore, the knowledge of risk factors that contribute to miscarriages because of chromosomal aberrations is vital to prevent spontaneous pregnancy losses after ART. Previous studies show that chromosome abnormalities in the fetuses or embryos significantly increase with maternal age [4]. The paternal influence of damaged chromatin is more prominent after zygotic transcriptional activation [5]. However, there is limited data regarding paternal characteristics that cause miscarriage because of chromosomal aberrations.

In terms of research that focuses on the effects of paternal age on their offspring, studies have often found conflicting results. Most analyses show that advanced age of the male partners is associated with lower pregnancy rate and a higher risk of early spontaneous pregnancy loss [6, 7]. Conflicting studies found that male partner age was not associated with adverse obstetrical or perinatal outcomes [2, 8]. Recent studies show that sperms of older men are associated with increased chromosomal abnormalities [9–13]. Older fathers transmit these genetic and chromosomal defects to their offspring and increase the incidence of miscarriages [14–16]. Moreover, advanced paternal age also significantly increases the risk of chromosomal aneuploidy in the embryos [17]. However, another study did not find male age to be linked to aneuploidy in the transferred embryos [8]. While these studies have looked at the effects of male age on the chromosomal abnormalities in the sperms or transferred embryos, very few studies have focused on the influence of paternal age on the chromosomal aberrations in miscarried fetuses.

The World Health Organization (WHO) published a global trend towards a decline in different sperm parameters in the last decades [18]. Sperms with poor viability affect embryo quality and continuing after embryo transfer, resulting in miscarriage in the early gestation period [4, 19, 20]. However, few studies have investigated the correlation between adverse sperm parameters and miscarriages related to chromosomal aberrations.

Recent estimates from the WHO suggest that more than 1.9 billion adults aged 18 years and older, were overweight worldwide [21]. High BMI is associated with poor sperm quality, low rates of blastocyst development, clinical pregnancy and live births, and higher rates of miscarriage [22–25]. Obesity in males is associated with epigenetic alterations in the sperm genomes, which adversely affects spermatogenesis, sperm function, embryo development, and health of the newborn offspring [26–29]. However, the relationship between paternal BMI and chromosomal abnormalities in the miscarried fetus remains inconclusive.

Expression of paternal genes in the chorionic villus samples (CVS) significantly correlate with fetal growth parameters during pregnancy [30]. Genome-wide testing of the miscarried chorionic villi using array-based single nucleotide polymorphisms (SNPs) has greatly contributed to understanding the etiology of spontaneous miscarriages due to genetic abnormalities [31]. In this study, we investigated the role of paternal characteristics such as age, BMI, and semen parameters in chromosomal aberrations-related miscarriage by analyzing the SNP array data from spontaneously miscarried CVS tissues.

## RESULTS

### Characteristics of study subjects

Table 1 shows the basic clinical characteristics of the male and female partners, sperm-related parameters, and the ART strategies. Supplementary Table 1 shows the comparison for different variables between younger (below 40 years) and older (40 years and above) male partner groups. All sperm samples were received as fresh, non-donor specimen. The highest numbers of male partners in this study belonged to the 30-34 year age group at 30.5%. After the age of 35 years, More than 50% of male partners had a BMI ≥25 kg/m^2^, which is considered overweight.

**Table 1:** Paternal, maternal, and ART characteristics.

**Characteristics**	**Paternal age (years)**					
	**20-**	**25-**	**30-**	**35-**	**40-**	**45-60**
No of the cases	30 (3.2)	238 (25.7)	282 (30.5)	212 (22.9)	125 (13.5)	38 (4.1)
**Paternal Characteristics**						
**BMI (kg/m^2^)**						
<25	18 (60.0)	138 (58.0)	144 (51.1)	92 (43.4)	59 (47.2)	16 (42.1)
≥25	12 (40.0)	99 (41.6)	135 (47.9)	120 (56.6)	66 (52.8)	22 (57.9)
Unknown	0 (0.0)	1 (0.4)	3 (1.1)	0 (0.0)	0 (0.0)	0 (0.0)
**Sperm concentration (10^6^ /ml)**						
<1	5 (16.7)	5 (2.1)	3 (1.1)	2 (0.9)	2 (1.6)	1 (2.6)
1-14.9	3 (10.0)	30 (12.6)	31 (11.0)	28 (13.2)	9 (7.2)	2 (5.3)
≥15	22 (73.3)	189 (79.4)	227 (80.5)	177 (83.5)	105 (84.0)	34 (89.5)
Unknown	0 (0.0)	14 (5.9)	21 (7.4)	5 (2.4)	9 (7.2)	1 (2.6)
**Sperm motility (%)**						
<10	3 (10.0)	7 (2.9)	4 (1.4)	4 (1.9)	4 (3.2)	1 (2.6)
10–39.9	3 (10.0)	30 (12.6)	43 (15.2)	54 (25.5)	31 (24.8)	13 (34.2)
≥40	24 (80.0)	197 (82.8)	232 (82.3)	152 (71.7)	89 (71.2)	24 (63.2)
Unknown	0 (0.0)	4 (1.7)	3 (1.1)	2 (0.9)	1 (0.8)	0 (0.0)
**Semen volume (ml)**						
<1.5	0 (0.0)	14 (5.9)	16 (5.7)	22 (10.4)	10 (8.0)	3 (7.9)
1.5–2.9	12 (40.0)	77 (32.4)	71 (25.2)	57 (26.9)	38 (30.4)	14 (36.8)
≥3	18 (60.0)	143 (60.1)	191 (67.7)	130 (61.3)	76 (60.8)	21 (55.3)
Unknown	0 (0.0)	4 (1.6)	4 (1.4)	3 (1.4)	1 (0.8)	0 (0.0)
**Sperm morphology (%)**						
<1	2 (6.7)	5 (2.1)	3 (1.1)	3 (1.4)	2 (1.6)	0 (0.0)
1-3.9	2 (6.7)	19 (8.0)	21 (7.4)	11 (5.2)	9 (7.2)	3 (7.9)
≥4	21 (70.0)	170 (71.4)	218 (77.3)	174 (82.1)	99 (79.2)	29 (76.3)
Unknown	5 (16.6)	44 (18.5)	40 (14.2)	24 (11.3)	15 (12.0)	6 (15.8)
**Maternal characteristics**						
**Age (years)**						
Mean (95% CI)	25.1 (24.3, 26.0)	27.8 (27.4, 28.1)	31.3 (31.0, 31.6)	35.5 (35.1, 35.9)	39.3 (38.7, 39.8)	40.5 (39.5, 41.5)
**BMI (kg/m^2^)**						
<25	23 (76.7)	179 (75.2)	189 (67.0)	158 (74.5)	90 (72.0)	26 (68.4)
≥25	7 (23.3)	59 (24.8)	93 (33.0)	54 (25.5)	35 (28.0)	12 (31.6)
**TSH (mIU/L)**						
Mean (95% CI)	2.35 (1.93, 2.78)	2.40 (2.26, 2.53)	2.46 (2.32, 2.60)	2.35 (2.20, 2.51)	2.31 (2.12, 2.50)	2.33 (1.97, 2.68)
**Prior births (full-term and pre-term)**						
0	30 (100)	222 (93.3)	231 (81.9)	130 (61.3)	69 (55.2)	17 (44.7)
1	0 (0.0)	16 (6.7)	48 (17.0)	75 (35.4)	53 (42.4)	20 (52.6)
≥2	0 (0.0)	0 (0.0)	3 (1.1)	7 (3.3)	3 (2.4)	1 (2.6)
**Prior miscarriages (spontaneous pregnancy loss)**						
0	27 (90.0)	198 (83.2)	228 (80.9)	149 (70.3)	80 (64.0)	27 (71.1)
1	3 (10.0)	33 (13.9)	37 (13.1)	47 (22.2)	32 (25.6)	8 (21.1)
≥2	0 (0.0)	7 (2.9)	17 (6.0)	16 (7.5)	13 (10.4)	3 (7.9)
**ART characteristics**						
**Fertilization method**					
IUI	7 (23.3)	23 (9.7)	23 (8.2)	18 (8.5)	7 (5.6)	2 (5.1)
IVF/ICSI	23 (76.7)	215 (90.3)	259 (91.8)	194 (91.5)	118 (94.4)	36 (94.7)
**No. of ovum pick-up**						
Median (interquartile range)	16 (12-26)	16 (11-21)	14 (9-19)	12 (8-18)	8 (5-13)	8 (4-14)
**Day of embryos transferred**						
Day3	12 (52.2)	130 (60.5)	171 (66.0)	152 (78.4)	100 (84.7)	27 (75.0)
Day5	11 (47.8)	85 (39.5)	88 (34.0)	42 (21.6)	18 (15.3)	9 (25.0)
**No. of embryos transferred**						
1	8 (34.8)	62 (28.8)	71 (27.4)	44 (22.7)	25 (21.2)	13 (36.1)
2	15 (65.2)	153 (71.2)	184 (71.0)	145 (74.7)	91 (77.1)	22 (84.6)
3	0 (0.0)	0 (0.0)	4 (1.5)	5 (2.6)	2 (1.7)	1 (2.8)
**Gestational age at miscarriage (weeks)**						
Median (interquartile range)	8.5 (8.0-10.0)	9.0 (8.0-9.0)	9.0 (8.0-9.0)	9.0 (8.0-9.0)	9.0 (8.0-9.0)	9.0 (8.0-9.3)

Data are presented as numbers (percentages), means (95% CI), or median (25^th^, 75^th^ percentile). BMI=body mass index; CI=Confidence interval; ART=assisted reproductive technology; TSH=thyroid stimulating hormone; IU=international unit; IUI=intrauterine insemination; IVF=in vitro fertilization; ICSI=intracytoplasmic sperm injection.

The mean age of female partners increased with advancing age of their male partners. In different paternal age groups, there were no significant differences in the female partner-related parameters such as BMI, TSH levels, fertilization method, gestational age at miscarriage (irrespective of the type of fertilization method), and the number of embryos transferred. The older couples showed higher percentage of prior births (56.5% vs. 19.5%; P<.001) and prior miscarriages (17.2% vs. 8.2%; P=.001) compared to the younger group. The median ovum pick-up numbers were higher in the younger group compared to the older group (younger: 14 ovum, range: 9-19; older: 8 ovum, range: 5-13; P<.001). The proportion of day 5 blastocysts transferred were higher for the younger group when compared with the older group (32.7% vs. 17.5%; P<.001).

### Results of the SNP array analysis on miscarried chorionic villi

**Figure 1:**
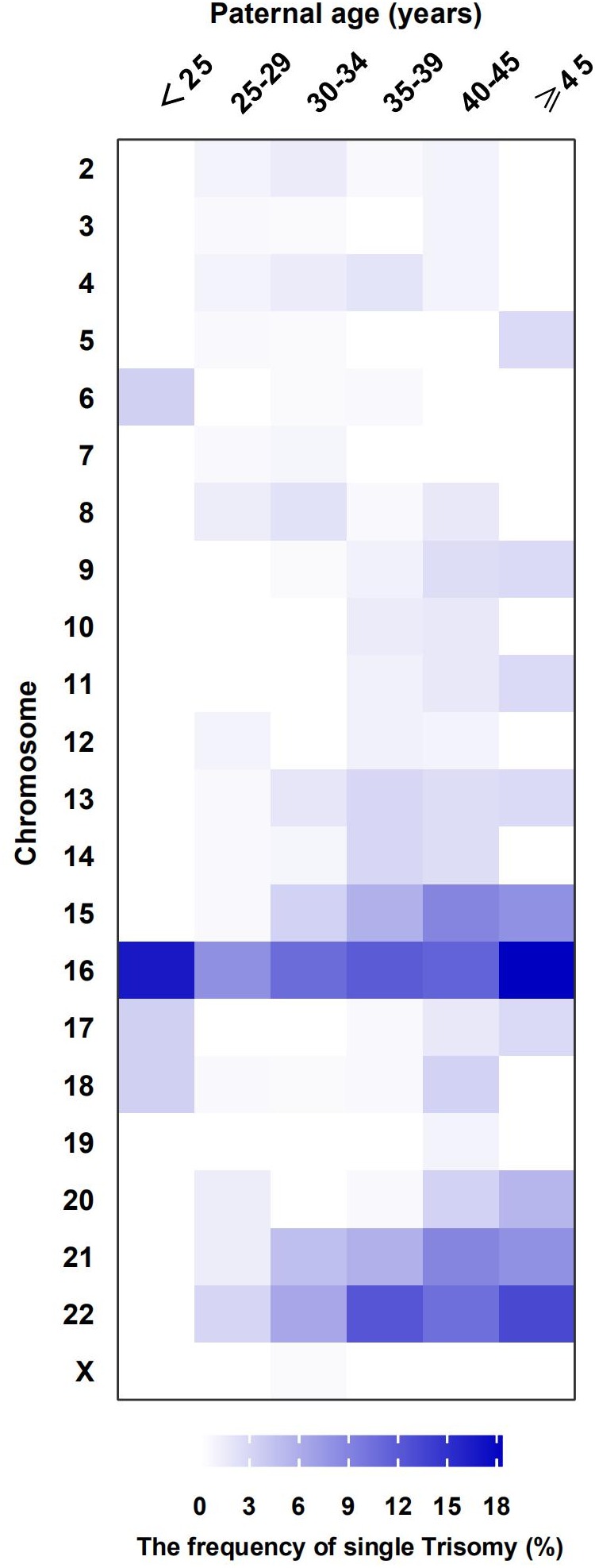
**The distribution of frequency in single Trisomy by paternal age groups.** Frequency of chromosomes (aneuploid: trisomy) in different paternal age groups are represented by a colour gradient, in the heat map.

Table 2 shows the frequencies of different abnormal chromosomal karyotypes in the miscarried fetuses between different paternal age groups were compared. The SNP array analysis shows abnormal karyotypes in 60.2% (557/925) of the chorionic villi from miscarriages. The most common abnormality was trisomy, accounting for 73.1% (407/557) of all identified abnormalities. Among the trisomy cases, 89.9% (366/407) were single chromosome trisomies and 5.2% (21/407) were multiple trisomies (where two or more chromosomes are involved). Microdeletions or microduplications were observed in the remaining 4.9% (20/407) of trisomy cases. Among the single-chromosome trisomies, the most prevalent was trisomy 16 (100/366; 27.3%), followed by trisomy 22 (69/366; 18.9%) and trisomy 21 (42/366; 11.5%). The prevalence of trisomy, especially in chromosomes 16, 22, and 21, increased with paternal age. However, it should be noted that the prevalence of trisomy 6, 16, 17, and 18 were observed a slighter increase for the youngest male age group (Figure 1).

**Table 2:** Spectrum of abnormal chromosomal karyotype in miscarried conceptus: type and frequency variations between paternal age groups.

**Variables**	**Paternal age (years)**		**Total frequency (%)**
	**20-**	**25-**		**30-**	**35-**	**40-**	**45-60**
Trisomy	73.1 (407/557)
Single	8	49	98	106	80	25	
Multiple	0	4	4	7	4	2	
Trisomy and duplication/deletion	1	3	5	6	5	0	
Monosomy	5.7 (32/557)
allosome	2	11	8	5	0	0	
autosome	0	2	1	1	2	0	
Structural abnormalities	9.0 (50/557)
Duplication or deletion	2	16	17	6	1	2	
Complex abnormalities	0	0	1	3	1	1	
Triploidy	2	12	11	4	4	0	5.9 (33/557)
Mosaicism	1	11	9	8	3	3	6.3 (35/557)
Total	16	108	154	146	100	33	–
Total frequency (%)	53.3 (16/30)	45.4 (108/238)	54.6 (154/282)	68.9 (146/212)	80.0 (100/125)	86.8 (33/38)	60.2 (557/925)

Values are numbers and frequencies.

**Figure 2:**
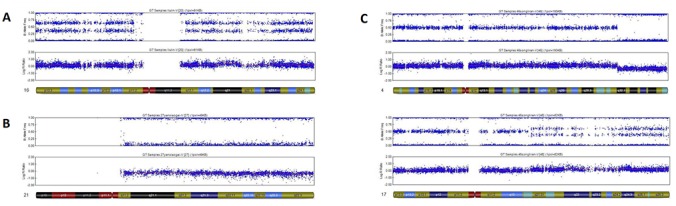
**Representative examples of SNP results.** (**A**) Single trisomy of chromosome 16. (**B**) Monosomy autosome (a monosomy of chromosome 21). (**C**) Complex abnormalities: Structural deletion with duplication.

Monosomy was found in 5.7% of samples (32/557), of which, 81.3% (26/32) were on the X-chromosome and 18.8% (6/32) were on autosomal chromosomes. Chromosomal structural abnormalities were found in 9.0% of the samples (50/557), of which, 88.0% (44/50) were duplications or deletions, and 12.0% (6/50) were complex abnormalities. Triploidy was found in 5.9% of samples (33/557) and mosaicism was found in 6.3% of samples (35/557). Representative examples of SNP results are shown in Figure 2.

### Paternal effects on the risk of chromosomal aberrations-related miscarriages

The proportion of abnormal karyotypes in the miscarried fetuses positively correlates with the age of male partners (linear by linear association=59.94; P<.001). However, the percentage of chromosomal abnormalities in abortuses were slightly higher for the youngest male partner group aged 20-24 years compared to male partners aged 25-29 years (53.3% vs. 45.4%). In addition, there were higher chromosomal aberration rates in miscarried fetuses for male partners BMI ≥25 kg/m^2^ and semen volume <1.5 ml (Supplementary Table 2). Univariate and multivariate analysis were used to determine potential risk factors for fetal development of chromosomal abnormalities (Table 3). Univariate analyses showed that in comparison to the control group of male partners aged 25-29 years, the risk of chromosomal abnormality-related miscarriage was significantly higher in all other higher age male partner groups, namely, above 45 years (odds ratio=7.94; 95% confidence interval, 3.00-21.05; P<.001), 40-44 years (4.82; 2.90-8.00; P<.001), 35-39 years (2.66; 1.81-3.92; P<.001), and 30-34 years (1.45; 1.02-2.05; P=.036). Moreover, parameters such as paternal BMI (P<.001), semen volume (P=.020), and maternal age (P<.001) significantly correlated with the chromosomal abnormality-related miscarriage.

**Table 3:** Logistic analyses of factors related to chromosomal aberration-related miscarriages.

**Variable**	**Univariable analysis**		**Multivariable analysis**	
	**Crude OR (95% CI)**	**P value**	**Adjusted OR (95% CI)**	**P value**
**Paternal age (years)**				
<25	1.38 (0.64, 2.95)	NS	2.48 (1.03, 5.96)	.042
25-29	1*		1*	
30-34	1.45 (1.02, 2.05)	.036	1.07 (0.68, 1.68)	NS
35-39	2.66 (1.81, 3.92)	.000	0.94 (0.51, 1.72)	NS
40-44	4.82 (2.90, 8.00)	.000	1.16 (0.50, 2.71)	NS
≥45	7.94 (3.00, 21.05)	.000	2.05 (0.56, 7.41)	NS
**Paternal BMI (kg/m^2^)**				
<25	1*		1*	
≥25	1.64 (1.26, 2.14)	.000	1.56 (1.14, 2.14)	.005
**Semen volume (ml)**				
<1.5	1.97 (1.11, 3.47)	.020	2.09 (1.06, 4.11)	.034
≥1.5	1*		1*	
**Sperm concentration (10^6^/ml)**				
<15	0.85 (0.58, 1.25)	NS	0.99 (0.61, 1.59)	NS
≥15	1*		1*	
**Sperm motility (%)**				
<40	1.04 (0.76, 1.44)	NS	0.83 (0.54, 1.28)	NS
≥40	1*		1*	
**Sperm morphology (%)**				
<4	0.69 (0.44, 1.10)	NS	0.77 (0.44, 1.37)	NS
≥4	1*		1*	
**Maternal age (years)**	1.13 (1.10, 1.17)	.000	1.13 (1.07, 1.20)	.000
**Maternal BMI (kg/m^2^)**				
<25	1*		1*	
≥25	0.92 (0.69, 1.24)	NS	0.95 (0.67, 1.34)	NS

Values are logistic regression odds ratios with 95% confidence intervals and P values.

*This variable functions as an indicator. Other categories of the same variable were compared with it. OR=odds ratio; CI=confidence intervals; NS=not significant; BMI=body mass index.

Multivariate analyses showed that the effect of male partner aged ≥30 was no longer statistically significant when they were adjusted for the age and BMI of female partners, and the BMI and semen parameters of the male partners. Interestingly, after adjustment, the youngest group in our study (fathers aged 20-24 years) showed a 148% higher risk of having a chromosomal abnormality-related miscarriage compared to the control group (adjusted odds ratio: 2.48, 95% confidence interval 1.03-5.96; P=.042). Furthermore, multivariate analyses showed that male partners with a high BMI (≥25 kg/m^2^) were at greater risk for chromosomal abnormality-related miscarriages compared to male partners with a lower BMI (adjusted OR=1.56; 95% CI: 1.14-2.14; P=.005). Furthermore, the risk of chromosomal abnormality-related miscarriages was higher for male partners with low semen volume (less than 1.5 ml) compared to those with higher semen volume (adjusted OR =2.09; 95% CI: 1.06-4.11; P=.034), whereas the other sperm parameters assessed, including sperm concentration, motility, and morphology were not significantly correlated with chromosomally abnormal abortus (P>.05). Moreover, our study was in agreement with the previous findings that advanced maternal age was a risk factor for chromosomal abnormality-related miscarriages (adjusted OR=1.13; 95% CI: 1.07-1.20; P<.001).

## DISCUSSION

Our study shows that none of the paternal groups aged more than 30 years significantly affect chromosomal aberrations-related miscarriages. Despite the effect we detected of male partners aged 30 years or older increasing the rate and relative risk of chromosomal aberrations-related miscarriages, none of these findings were found to be statistically significant once adjusting for covariates known to affect karyotypes in fetuses.

However, after adjustment, the risk of abnormal karyotypes in miscarried fetuses was higher for the youngest male partner group aged 20-24 years compared to male partners aged 25-29 years. The reasons for this finding need to be investigated in the future studies, but, we speculate that there are two reasons for this result in this study. The first reason is that the sample size of the youngest age group in this study is less than one hundred cases, which may lead to the bias of the result due to the small sample size. The second possible reason is the immaturity of the reproductive system at a very young age. Our study also demonstrates that elevated BMI and low semen volume of the male partner significantly increased the risk of chromosomal abnormalities in the miscarried fetuses, while neither sperm concentration, motility, nor morphology was associated with chromosomal aberrations-related miscarriages.

Previous studies have shown that male aging increases genomic instability in the sperm DNA in the form of epigenetic changes, DNA fragmentation, telomere length reduction, gene mutations, and other chromosomal aberrations [9–13]. Paternal age was also associated with increased aneuploidy of chromosomes and mosaicism in both cleavage and blastocyst stage embryos [17]. However, another study did not find male age to be linked to aneuploidy in the transferred embryos [8]. Differing from these studies that looked at the effects of male age on the chromosomal aberrations in the sperms or transferred embryos, we focus mainly on the effect of paternal age on abnormal chromosomes in miscarried fetuses. We believe this current research adds another helpful layer regarding the association between paternal age and fetal development. Our study demonstrates that advancing male partner age did not alter the incidence of chromosomal abnormalities-related miscarriages when adjustment was made for female recipients age and BMI, as well as male partners BMI and sperm parameters. However, the youngest male partner group aged 20-24 years shows higher risk of abnormal chromosomes in miscarried fetuses compared to the control group.

A study by Capelouto et al. found no association between worsening sperm parameters and rates of clinical pregnancy, miscarriage, and live births [2]. On the contrary, Lee et al. showed that poor sperm parameters correlated with lower pregnancy and higher miscarriage rates [19]. Furthermore, poor sperm quality is associated with decreased blastocyst formation and increased sex chromosome aneuploidy in embroys [20]. However, Mazzilli et al. reported that sperm parameters were not linked to aneuploidy in the transferred embryos [8]. Our study further investigates the influence of sperm parameters on chromosomal karyotype in abortuses which were after the embryos transferred and demonstrates that low semen volume significantly increased the risk of miscarriages because of chromosomal abnormalities in fetuses.

The mechanistic link between elevated BMI and higher chromosomal abnormalities in miscarried fetuses remains elusive. Recent studies show that male partners with high BMIs have altered sperm epigenetic patterns [27, 29]. The epigenetic marks represent important regions in sperm DNA that regulate embryonic development and embryo quality [28, 32]. Moreover, specific microRNAs and other non-coding RNAs play a vital role in normal embryogenesis [33]. Our study demonstrates that elevated BMI in male partners negatively impacts chromosomal karyotype in miscarried fetuses.

Our study has several strengths and limitations. First to reduce the effects of confounding factors, we excluded male partners with abnormal karyotypes, congenital and/or chronic diseases, and azoospermia. Second, we performed multivariate regression analysis after adjusting for potential confounding maternal characteristics, including female recipient age and BMI. Finally, to our knowledge, this is the first report regarding the effects of male partner characteristics on chromosomal abnormalities-related miscarriage.

There are several limitations in the use of electronic medical record database for such studies. While the ART data was reviewed for errors after being completed by parents and healthcare workers, further inaccuracies cannot be ruled out. Furthermore, the database did not record environmental and occupational information, as well as, unhealthy lifestyle and psychological status of patients, which may contribute to the miscarriage.

In conclusion, our study shows that the youngest male partner group aged 20-24 years shows higher risk of abnormal chromosomes in the miscarried fetuses compared to male partners group aged 25-29 years, while the other male partners groups aged 30 years or older have negligible effects on chromosomal aberrations-related miscarriages. Our study also demonstrates that decreased semen volume and elevated BMI of male partners is associated with increased risk of chromosomal aberrations-related miscarriages. Therefore, it is probable that we may establish protocols focusing on male weight loss before conception to reduce chromosomally abnormal miscarriage. Furthermore, our study suggests that male partners with very young age, high BMI or poor semen volume might need pre-implantation genetic screening (PGS) to identify any chromosomal abnormalities that may indicate a risk of miscarriage. Further large-scale multicenter clinical studies are necessary to confirm our findings.

## MATERIALS AND METHODS

### Study cohort and inclusion criteria

We conducted this retrospective analysis using data from the Clinical Reproductive Medicine Management System/Electronic Medical Record Cohort Database (CCRM/EMRCD), obtained from the Reproductive Medical Center, First Affiliated Hospital of Zhengzhou University, and Henan Province Key Laboratory for Reproduction and Genetics. This study was approved by our Hospital Ethics Committee. We obtained written informed consent from all patients during the first consultation.

**Figure 3:**
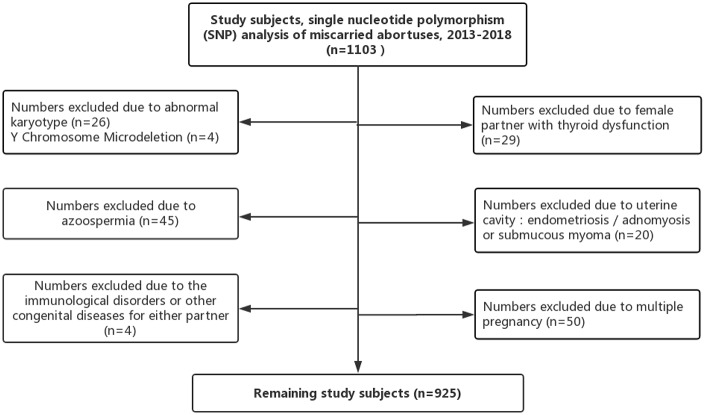
**Study inclusion and exclusion.**

The database included clinical information regarding 1103 patients who experienced involuntary miscarriage between January 2013 and December 2018 in our fertility clinic after ART. The patients sent their miscarried fetuses to our pre-implantation genetic diagnosis center for analysis. As shown in Figure 3, we excluded patients with abnormal chromosome karyotype for either partner (30 patients), uterine factors such as endometriosis, adenomyosis or submucous myoma (20 patients), female partner with thyroid dysfunction (29 patients), multiple pregnancy (50 patients), immunological disorders or other congenital diseases for either partner (4 patients), non-obstructive or obstructive azoospermia (45 patients), and factors related to infectious agents (0 patients). We obtained all clinical information of the study couples, including the ART process that they underwent and evaluated the relationship between miscarriage due to chromosomal abnormalities and paternal characteristics such as paternal age, BMI, and semen parameters.

### ART process

Among the study participants, 80 underwent intrauterine insemination (IUI), 642 received *in vitro* fertilization (IVF), and 203 received intracytoplasmic sperm injections (ICSI) as described previously [34]. The serum β-hCG levels were monitored on days 14 and 18. Clinical pregnancy was confirmed when a gestational sac with a fetal heartbeat was detected by the ultrasound in the uterine cavity. Spontaneous miscarriage was defined as the absence of fetal cardiac pulsation in the uterine cavity after confirmation of clinical pregnancy. Once miscarriage was confirmed, the doctors performed curettage of the uterine cavity and the chorionic villi was sent to the pre-implantation genetic diagnosis center for analysis to identify the chromosomal abnormality that resulted in the adverse reproductive outcome.

### SNP array analysis

The chorionic villi were thoroughly separated from the maternal deciduas as previously published to avoid maternal genome contamination [35]. Total DNA from the chorionic villi was extracted using the All Prep DNA Mini Kit (Qiagen, Germany) and subjected to SNP array using the Human CytoSNP-12v.21 Array (Illumina, San Diego, California, USA). The SNP array data was analyzed using Genome-Studio (Illumina 2011) and Karyo-Studio v1.4 (Illumina 2011). The copy number variants (CNVs) were mapped using the DGV database (http://dgv.tcag.ca/dgv/app/faq) to identify the candidate pathogenic CNVs. All the steps were analyzed independently by at least two expert technicians.

### Statistical analysis

We reported the characteristics of all study couples (male partners and female recipients) and the ART variables. Continuous variables with normal distribution were represented as means and 95% confidence interval, and the differences between groups were analyzed using Student’s t-tests or one-way ANOVA. Continuous variables with skewed distribution were represented as median and interquartile range, and the differences between groups were analyzed using the Wilcoxon two-sample test. Categorical data were expressed as frequencies and percentages, and the differences between groups were analyzed using the Chi-square test. The numbers and percentages of abnormal chromosomal karyotypes for each paternal age group were compared using the chi-square test for trends to confirm if chromosomal aberration rate changed with paternal age.

Logistic regression analysis was performed to identify risk factors for chromosomal aberrations-related miscarriage. The variables analyzed include male partner age, BMI, sperm concentration, motility, morphology, semen volume, female recipient age, and BMI. Embryo transfer information was not included in the multivariable analysis because some study participants were treated with IUI. Multivariable analysis was carried out to determine the role of male partner age, BMI, and semen parameters on miscarriage due to chromosomal abnormalities. We categorized 925 patients into 6 age groups based on paternal age, namely, 20-24, 25-29, 30-34, 35-39, 40-44, and 45-60 years. The control group for the logistic regression models was 25 to 29 years male partner’s age group. The control group for BMI was the male BMI <25 kg/m^2^ group, according to the World Health Organization definition of normal body weight [21]. The control groups for various sperm parameters were as defined by the 2010 World Health Organization reference values for human semen characteristics: sperm concentration ≥15 million/mL, motility ≥40%, morphologically normal forms ≥4%, and semen volume ≥1.5 mL [18]. Although there is no currently accepted medical definition for advanced paternal age, study show that the risk of miscarriage was highest for couples with maternal age ≥35 years and paternal age ≥40 years [36]. Thus, we defined male partner age ≥40 years as advanced paternal age in our study. The statistical analyses were performed using the IBM SPSS Statistics 25 software (IBM Corporation). All statistical tests were two sided and 95 percent confidence intervals were used to define the range of estimates.

## Supplementary Material

Supplementary Tables
