# Supplementary material for: Paternal age, body mass index, and semen volume are associated with chromosomal aberrations-related miscarriages in couples that underwent treatment by assisted reproductive technology
Source: Aging (Albany NY). 2020 May 8;12(9):8459–72. doi: 10.18632/aging.103151 (PMC7244044; doi:10.18632/aging.103151)
Supplement: Supplementary Tables [file aging-12-103151-s001..pdf]

SUPPLEMENTARY TABLES

Supplementary Table 1. Paternal, maternal, and ART characteristics between younger and advanced age fathers.

Characteristics	Younger group<br>Paternal age <40 years (n=762)	Older group<br>Paternal age ≥40 years (n=163)	P value
**Paternal Characteristics**			
BMI (kg/m^2)			NS
<25	392 (51.7)	76 (46.6)	
≥25	366 (48.3)	87 (53.4)	
**Semen volume (ml)**			NS
<1.5	52 (6.9)	13 (8.0)	
≥1.5	699 (93.1)	149 (92.0)	
**Sperm concentration (x10^6/ml)**			NS
<15	107 (14.8)	14 (9.2)	
≥15	615 (85.2)	139 (90.8)	
**Sperm morphology (%)**			NS
<4	66 (10.2)	14 (9.9)	
≥4	583 (89.8)	128 (90.1)	
**Sperm motility (%)**			.003
<40	148 (19.7)	49 (30.2)	
≥40	605 (80.3)	113 (69.8)	
**Maternal characteristics**			
**Age (years)**			
Mean (95% CI)	31.1 (30.8, 31.5)	39.5 (39.1, 40.0)	.000
**BMI (kg/m^2)**			NS
<25	549 (72.0)	116 (71.2)	
≥25	213 (28.0)	47 (28.8)	
**TSH (mIU/L)**			
Mean (95% CI)	2.40 (2.32, 2.49)	2.30 (2.13, 2.47)	NS
**Prior births (full-term and pre-term)**			.000
0	613 (80.4)	71 (43.6)	
1	139 (18.2)	86 (52.8)	
≥2	10 (1.3)	6 (3.7)	
**Prior miscarriages (spontaneous pregnancy loss)**			.001
0	700 (91.9)	135 (82.8)	
1	53 (7.0)	26 (16.0)	
≥2	9 (1.2)	2 (1.2)	
**ART characteristics**			
Fertilization method			NS
IUI	71 (9.3)	9 (5.5)	
IVF/ICSI	691 (90.7)	154 (94.5)	
**No. of oocytes pick-up**			
Median (interquartile range)	14 (9-19)	8 (5-13)	.000
**Day of embryos transferred**			.000
Day3	465 (67.3)	127 (82.5)	
Day5	226 (32.7)	27 (17.5)	
No. of embryos transferred			NS
1	185 (26.8)	38 (24.7)	

2	497 (71.9)	113 (73.4)	
3	9 (1.3)	3 (1.9)	
Gestational age at miscarriage (weeks)			
Median (interquartile range)	9 (8-9)	9 (8-9)	NS

Data are presented as numbers (percentages), means (95% CI), or median (25th, 75th percentile). BMI=body mass index; CI=Confidence interval; ART=assisted reproductive technology; TSH=thyroid stimulating hormone; IU=international unit; IUI=intrauterine insemination; IVF=in vitro fertilization; ICSI=intracytoplasmic sperm injection; NS=not significant.

Supplemental Table 2. The rates of the SNP array analysis comparing male partners BMI and sperm parameters.

Variable	Normal karyotype, n (%)	Abnormal karyotype, n (%)
**Paternal BMI (kg/m^2)**		
<25	213 (45.5)	255 (54.5)
≥25	153 (33.8)	300 (66.2)
**Semen volume (ml)**		
<1.5	17 (26.2)	48 (73.8)
≥1.5	348 (41.0)	500 (59.0)
**Sperm concentration (10^6/ml)**		
<15	53 (43.8)	68 (56.2)
≥15	300 (39.8)	454 (60.2)
**Sperm motility (%)**		
<40	77 (39.1)	120 (60.9)
≥40	288 (40.1)	430 (59.9)
**Sperm morphology (%)**		
<4	38 (47.5)	42 (52.5)
≥4	274 (38.5)	437 (61.5)

Values are numbers (percentages). SNP=single nucleotide polymorphism; BMI=body mass index.
